# A Markerless 2D Video, Facial Feature Recognition–Based, Artificial Intelligence Model to Assist With Screening for Parkinson Disease: Development and Usability Study

**DOI:** 10.2196/29554

**Published:** 2021-11-19

**Authors:** Xinyao Hou, Yu Zhang, Yanping Wang, Xinyi Wang, Jiahao Zhao, Xiaobo Zhu, Jianbo Su

**Affiliations:** 1 Department of Automation Shanghai Jiao Tong University Shanghai China; 2 Department of Neurology Xinhua Hospital Affiliated to Shanghai Jiao Tong University School of Medicine Shanghai China; 3 Department of Neurology Second Affiliated Hospital of Jiaxing City Jiaxing China; 4 Department of Neurology Shanghai East Hospital School of Medicine, Tongji University Shanghai China

**Keywords:** Parkinson disease, facial features, artificial intelligence, diagnosis

## Abstract

**Background:**

Masked face is a characteristic clinical manifestation of Parkinson disease (PD), but subjective evaluations from different clinicians often show low consistency owing to a lack of accurate detection technology. Hence, it is of great significance to develop methods to make monitoring easier and more accessible.

**Objective:**

The study aimed to develop a markerless 2D video, facial feature recognition–based, artificial intelligence (AI) model to assess facial features of PD patients and investigate how AI could help neurologists improve the performance of early PD diagnosis.

**Methods:**

We collected 140 videos of facial expressions from 70 PD patients and 70 matched controls from 3 hospitals using a single 2D video camera. We developed and tested an AI model that performs masked face recognition of PD patients based on the acquisition and evaluation of facial features including geometric and texture features. Random forest, support vector machines, and k-nearest neighbor were used to train the model. The diagnostic performance of the AI model was compared with that of 5 neurologists.

**Results:**

The experimental results showed that our AI models can achieve feasible and effective facial feature recognition ability to assist with PD diagnosis. The accuracy of PD diagnosis can reach 83% using geometric features. And with the model trained by random forest, the accuracy of texture features is up to 86%. When these 2 features are combined, an F1 value of 88% can be reached, where the random forest algorithm is used. Further, the facial features of patients with PD were not associated with the motor and nonmotor symptoms of PD.

**Conclusions:**

PD patients commonly exhibit masked facial features. Videos of a facial feature recognition–based AI model can provide a valuable tool to assist with PD diagnosis and the potential of realizing remote monitoring of the patient’s condition, especially during the COVID-19 pandemic.

## Introduction

Parkinson disease (PD) is a typical movement disorder, and its high rate of disability seriously affects the daily life of patients [1]. However, there is a lack of reliable methods for the early diagnosis of PD. The diagnostic accuracy of the clinical diagnosis of PD has been reported at 73.8% when performed mainly by nonexperts and 79.6% when performed by movement disorder experts [2]. Hence, it is important to develop a valuable tool to diagnose PD as early as possible. In recent years, computer technology and artificial intelligence (AI) have been making great advances in the diagnosis of PD. Many studies have been carried out by using computer technology from different aspects [3], including using wearable devices to study the gait of patients with PD [4] and using intelligent pens to study the trembling of patients’ writing based on their hand tremor [5]. However, the accuracy of these studies is not sufficient, and they cannot be applied on a large scale due to the limitations of equipment and technology.

Patients with PD usually suffer from loss of facial expressions. Their facial muscle movements and amplitude of expression are usually different from healthy people because of facial muscle stiffness, which is called masked face [6]. Based on this, research on the facial expressions of PD when watching videos and during social interactions has been reported [7]. The overall expressivity of PD has been verified as being reduced, but subjective evaluations from different clinicians often show low consistency [8]. And traditional methods are not accessible and fast enough for large-scale work. Computer vision has been considered to assist the work.

The use of 2D video to assist with medical diagnosis has long-standing precedents. In 2014, researchers used independent component analysis and a number of different classifiers to describe local shape variations and differentiate people with Down syndrome from the general population [9]. Basel-Vanagaite et al [10] used novel facial dysmorphology analysis to identify patients with Cornelia de Lange syndrome and achieved a good effect, with a recognition rate of 87%. Studies on facial expression recognition algorithms have also emerged in psychiatry [11]. These applications all show that 2D video is effective for diagnosis when the disease has an impact on facial expressions. The use of computer vision on prescreening can not only quickly process a large amount of data and reduce the burden on doctors but also provide reliable support to patients remotely. Now that the COVID-19 pandemic is ravaging, guidelines for avoiding contact can undoubtedly better support epidemic prevention and control. Therefore, we aimed to explore a convenient and accessible PD diagnostic method using a markerless 2D video, facial feature recognition–based, AI model.

In our study, the facial features of PD patients and nonpatients were collected that represent the speed, elasticity, and coordination of the facial muscles [12]. Our goal was to identify features from facial information that distinguish PD from healthy people and finally develop a markerless 2D video, facial feature recognition–based, AI model to assist with PD diagnosis.

## Methods

### Study Design and Participants

We performed a multicenter, observational, outpatient-based, cross-sectional study, which was approved by the Research Ethics Committee of Xin Hua Hospital affiliated with Shanghai Jiao Tong University School of Medicine and the Research Ethics Committee of each site of the study group. After obtaining informed consent, participants were consecutively recruited. PD was diagnosed according to the Movement Disorder Society PD criteria. Patients with secondary parkinsonism, stroke, brain tumor, or an alternative cause for parkinsonism symptoms were excluded. For the control group, age- and gender-matched healthy participants were recruited. The sample size of our study was determined using a priori statistical power analysis. On the basis of the literature, we predicted a large between-subjects effect size in our design using analysis of variance (ANOVA; *F*=0.4). A power analysis using G-Power indicated that a total sample size of 44 (22 per group) would be needed, with 95% power (1-β), an alpha of .05, and a correlation of 0.5 among the 120 repeated measurements. These analyses suggest that our recruited sample of 140 participants (70 patients, 70 controls) would be sufficient.

### Data Collection

A total of 70 consecutive PD patients (male/female: 33/37) and 70 matched controls (MC; male/female: 39/31) were included in our study. In this study, a structured interview for clinical and demographic variables was performed. All PD participants were evaluated during the medication “on” period. The following demographic and clinical feature data were obtained from the participants: gender, age, education, age at PD onset, duration of disease, the history of dopamine replacement therapy and related complications (total levodopa equivalent daily dose [LEDD] was calculated according to previously suggested conversion formulae), Hoehn-Yahr (H-Y) stage, Unified Parkinson Disease Rating Scale (UPDRS), Mini-Mental State Examination (MMSE), a scale for freezing of gait, Non-Motor Symptoms Scale (NMSS), REM Sleep Behavior Disorder Questionnaire Hong Kong (RBDQ-HK), Hamilton Anxiety Scale (HAM-A), Parkinson Disease Questionaire-39 (PDQ-39), and the Questionnaire for Impulsive-Compulsive Disorders in Parkinson disease (QUIP).

**Figure 1:**
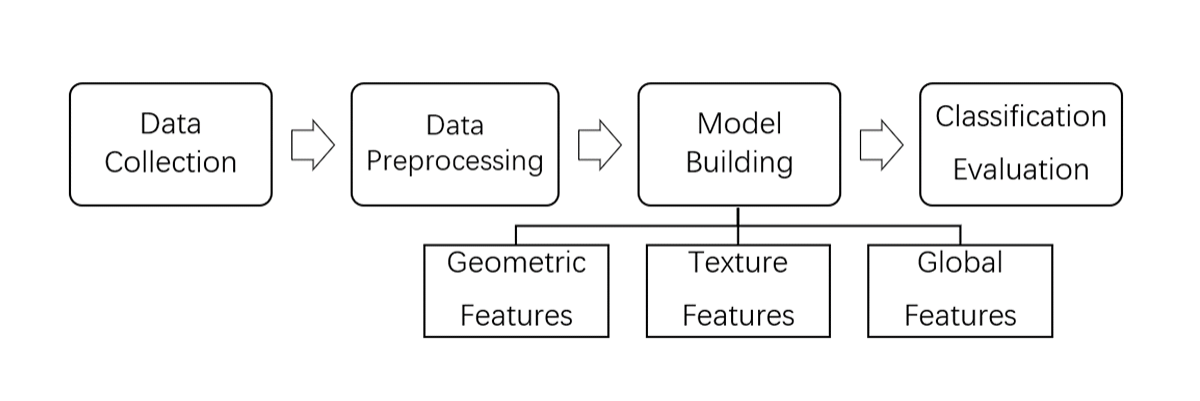
Flowchart of the research process.

The flowchart of the research process is shown in Figure 1 and included the following steps: Collect a series of facial expressions from patients and nonpatients, and perform a series of preprocessing operations to improve the quality of the samples. The corresponding models were constructed by extracting geometric features and texture features. Finally, these 2 features were combined as combined features to establish a patient identification model of PD, and the classification results of the model were evaluated.

The collected videos lasted 10-15 seconds each, and the patient involved was instructed to make certain expressions, including poker face and smiling, and was recorded by the camera. For each participant, we collected one such corresponding video.

### Preprocessing of Facial Images

The main work of preprocessing was the extraction of facial areas and localization and normalization of facial key points. Since information for facial expressions would be used, the ideal output of the preprocessing was the pure expressions. The preprocessing of facial images for this paper included the following 3 steps: (1) identify the existing face in the sample picture, and select the recognized face with a rectangular box; (2) locate the facial feature points in the intercepted rectangular box using 68 feature points, based on ensemble of regression trees cascade regression [13], to describe the position relationship of each key part of the face in detail; (3) use an affine transformation matrix to normalize the face to achieve the goal of face correction, which was ​​obtained by the corresponding coordinate relationship between the feature points. In our study, face recognition and facial landmark detection were realized based on Dlib, a machine learning, open-source library. The preprocessing of sample images was processed through this series of processes to obtain the normalized facial image.

### Extracting Facial Geometric Features

**Figure 2:**
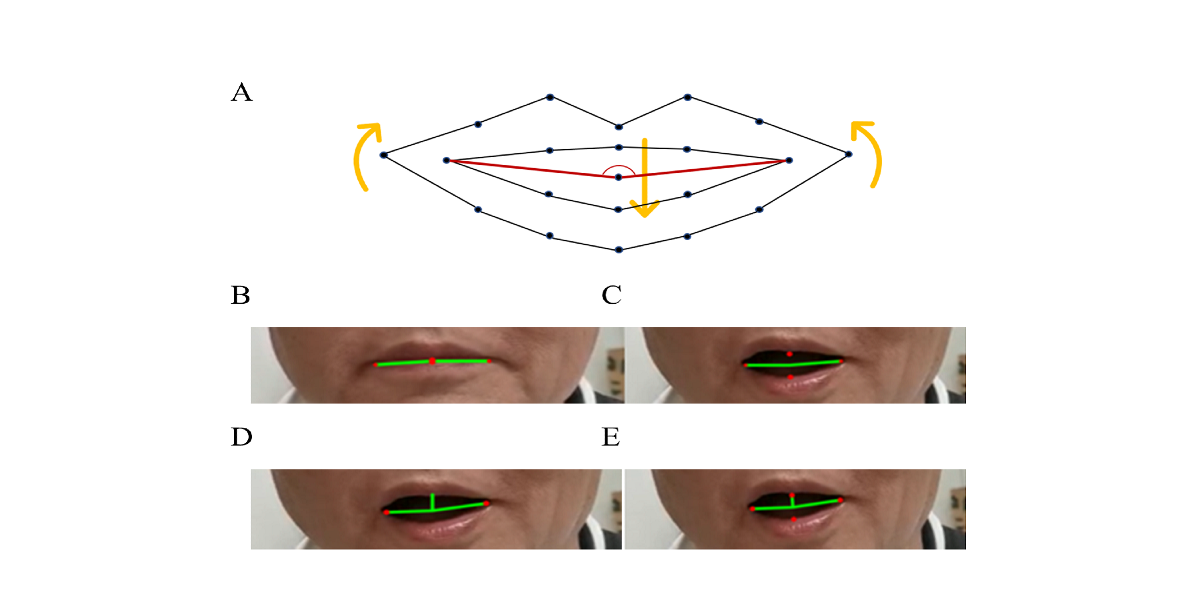
The characteristic angle of the mouth. (A) movement and the relative position of key points on the mouth. (B) features of the angle before smiling. (C) features of the angle after smiling. (D) deviation angle of the overall mouth after smiling. (E) deviation angle of the left and right sides of the mouth after smiling.

In this section, features were extracted from specific frames of the collected video, during which patients were poker-faced or smiling. The difference between these 2 frames was considered as features for further use. Due to the presence of “masked face” features in PD and the obvious appearance in the area of the mouth, the characteristic angle of the mouth was extracted using the key points of the mouth. Figure 2A shows the key points of the mouth and their movement direction during a smile, as well as the marking number of each key point in the detection process. The red lines construct the characteristic triangle of the mouth in this paper.

**Figure 3:**
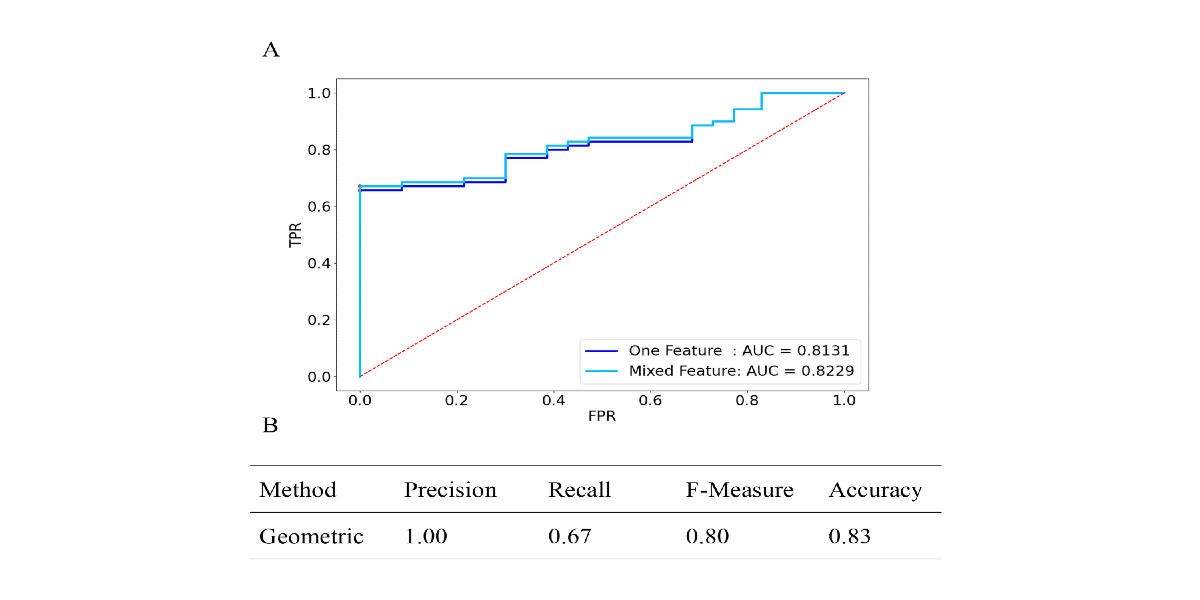
Receiver operating characteristics (ROC) analysis for the diagnosis of Parkinson disease using geometric feature and the recognition result. (A) ROC curve for each parameter and (B) result of machine learning algorithms. AUC: area under the curve; FPR: false positive rate; TPR: true positive rate.

We defined 3 feature angles according to these feature points: (1) the angles before and after laughter, as shown in Figure 2B and Figure 2C; (2) the overall deviation angle of the mouth, as shown in Figure 2D; (3) the deviation angle of the left and right sides of the mouth, as shown in Figure 2E.

The feature angles constructed in our study can represent the overall range of motion of the mouth, so that the difference between the feature angle before and after a smile can reflect the stiffness of the mouth muscles in patients with PD, which can be used as a main feature. At the same time, the overall deviation angle of the mouth and the deviation angles of the left and right sides of the mouth can fully reflect the mouth asymmetry caused by uncoordinated mouth muscle movements in some patients with PD, so these geometric features can be used as an auxiliary feature. Thus, these features were mixed to construct a model to differentiate patients with PD by using geometric features.

### Extracting Facial Texture Features

Compared with geometric features, texture features retain more important details because of the multiple dimensions. Moreover, facial texture is a relatively intuitive existence. For different people, texture itself has distinct characteristics, which can be expressed through features. In our study, the histogram of oriented gradient (HOG) [14,15] and local binary pattern (LBP) features [16,17] were used to describe facial textures. First, all samples were preprocessed. In order to further reduce the influence of irrelevant factors, the mouth and eye regions were selected as the feature regions and extracted for feature extraction. HOG features and LBP features were extracted from the obtained feature region [18].

For classification of texture features, this study used support vector machine (SVM) [19,20], k-nearest neighbor (KNN) [21,22], and random forest (RF; Tree) [23,24] to establish a recognition model to distinguish patients from people without PD. And we compared the results obtained by the different classifier algorithms to verify that the texture features of PD patients’ faces are indeed different from those of MCs [25].

### Statistical Analysis

All characteristics of our study were summarized using means and SDs for continuous variables and percentages and frequencies for categorical variables. The comparisons were performed using Student *t* tests to analyze continuous variables with parametric distributions and Mann-Whitney U tests to test variables with nonparametric distributions. And the Pearson chi-square test was used for categorical variables. Logistic regression analyses were adjusted for gender, age, age at PD onset, disease duration, and educational level. The level of significance was set at *P*<.05. Odds ratios (ORs) are presented with their 95% CIs. We used receiver operating characteristic (ROC) [26] analysis to assess the effectiveness of the classification. For the statistical analysis and to generate graphs, SPSS v24 (IBM Corp, Armonk, NY) and Prism 8.0 for Windows (GraphPad Software Inc, San Diego, CA) were used.

## Results

### Geometric Features of PD

For the geometric features, the samples were divided into a training set containing 80 sets of data and a testing set containing 60 sets of data. The area under the curve (AUC) of the model was 0.8131 for the main feature and 0.8229 for the mixed feature; values above 80% mean it has good performance for this problem (Figure 3A). The best threshold, Youden index from the ROC curve, and relevant F-measure and other indicators are shown in Figure 3B. The overall recognition effect of geometric features was about 80%, and the precision was 100%, while the recall was only 67%. However, the geometric feature model was not associated with clinical characteristics of PD (Table 1).

**Table 1:** Correlations between clinical characteristics of Parkinson disease (PD) and geometric features of the face.

Variable	*P* value	r	95% CI
Age	.28	–0.131	–0.355 to 0.108
Age at onset	.08	–0.212	–0.426 to 0.024
PD duration	.12	0.19	–0.048 to 0.406
DALEDD^a^	.12	0.188	–0.049 to 0.405
QUIP^b^	.35	–0.114	–0.34 to 0.124
HAM-A^c^	.38	0.106	–0.132 to 0.333
RBD^d^	.53	–0.076	–0.305 to 0.0162
Freezing of gait	.73	0.042	–0.195 to 0.274
Total UPDRS^e^	.31	0.123	–0.115 to 0.348
HY^f^	.46	0.089	–0.149 to 0.318
MMSE^g^	.15	0.172	–0.065 to 0.391
NMSS^h^	.06	–0.223	–0.436 to 0.012
PDQ-39^i^	.54	0.074	–0.164 to 0.303

^a^DALEDD: levodopa equivalent daily doses.

^b^QUIP: Questionnaire for Impulsive-Compulsive Disorders in Parkinson disease.

^c^HAM-A: Hamilton Anxiety Scale.

^d^RBD: REM Sleep Behavior Disorder.

^e^UPDRS: Unified Parkinson Disease Rating Scale.

^f^HY: Hoehn & Yahr.

^g^MMSE: Mini-Mental State Examination.

^h^NMSS: Non-Motor Symptoms Scale.

^i^PDQ-39: Parkinson Disease Questionaire-39.

### Texture Features of PD

**Figure 4:**
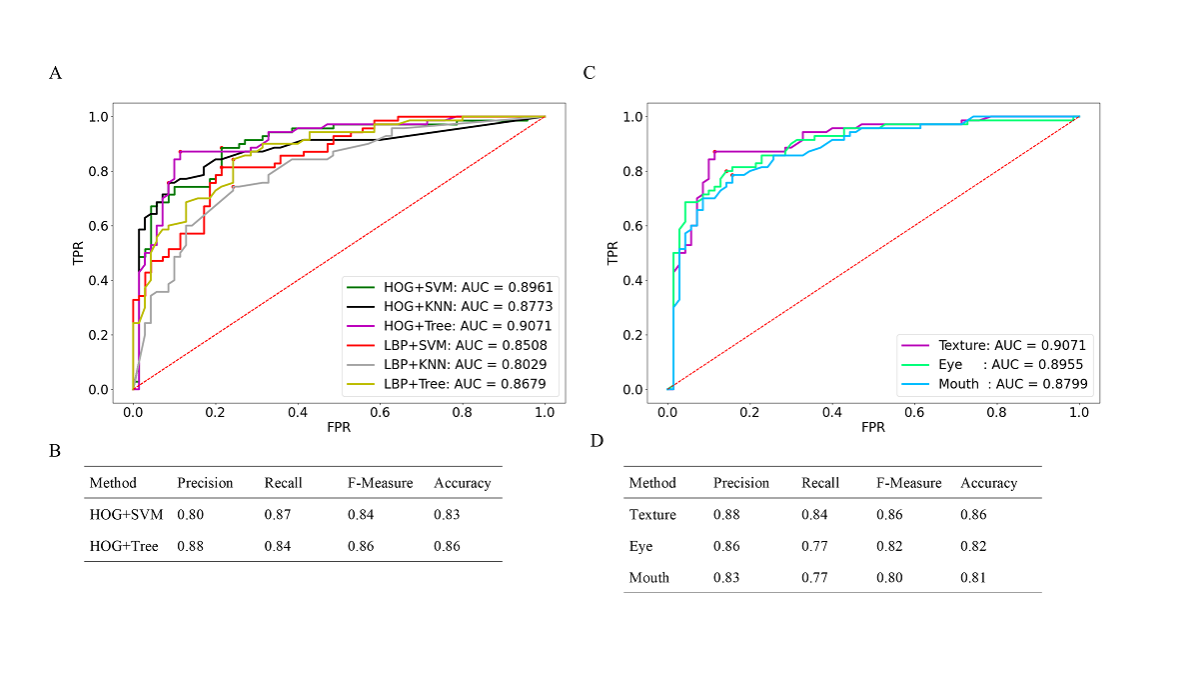
Receiver operating characteristics (ROC) analysis for the diagnosis of Parkinson disease using texture features and the recognition result. (A) ROC curve for 2 texture feature extraction algorithms with 3 classification models and (B) best result of machine learning algorithms. (C) ROC curve for texture features of the region of the eye, region of the mouth and their combination and (D) result of machine learning algorithms. AUC: area under the curve; FPR: false positive rate; HOG: histogram of oriented gradient; KNN: k-nearest neighbor; LBP: local binary patern; SVM: support vector machine; TPR: true positive rate.

For the texture features, the AUC for LBP+KNN achieved 0.8029. The HOG+SVM (AUC=0.8961) and HOG+Tree (AUC=0.9071) methods performed the best. Regarding the texture feature extraction algorithms, the comprehensive performance of HOG was better than that of LBP, as the AUCs of HOG were all greater than 0.8773 (Figure 4A). The best threshold, Youden index from ROC curve, and relevant F-measure and other indicators are shown in Figure 4B. Because of the similar AUC results of HOG+SVM and HOG+Tree, we report the results from both. The overall recognition effect of HOG+Tree could reach 0.86, which means this method has excellent performance for this identification problem. While the recall of HOG+SVM was 0.87, most of the PD patients could be recognized under this method. And the F-measure of the methods was approximately 85%.

Since HOG+SVM and HOG+Tree performed well and had similar results, we further examined whether these texture features were significantly correlated with the clinically evaluated variables from the PD patients. It can be seen from Table 2 that the results of the texture features model were not associated with motor and nonmotor symptoms in PD.

To further explore the differences between the features of the eye and mouth in PD, we preprocessed the eye and mouth regions and obtained the corresponding texture features. Then, we compared the results of the diagnosis using the HOG+Tree method to explore the differences between them. It can be seen from Figure 4C that the AUC of the eye was 0.8955, which is higher than the 0.8799 of the mouth. And the values for the eye are better than those for the mouth for other evaluation indices (Figure 4D).

**Table 2:** Correlations between the clinical characteristics and 2 main texture features.

Variable	HOG^a^+SVM^b^			HOG+Tree^c^		
	*P* value	r	95% CI	*P* value	r	95% CI
Age	.21	–0.152	–0.373 to 0.086	.13	–0.184	–0.402 to 0.053
Age at onset	.15	–0.175	–0.394 to 0.063	.26	–0.136	–0.359 to 0.102
PD^d^ duration	.72	–0.043	–0.275 to 0.194	.15	–0.175	–0.394 to 0.062
DALEDD^e^	.75	0.039	–0.198 to 0.272	.65	–0.055	–0.286 to 0.183
QUIP^f^	.22	0.15	–0.088 to 0.372	.18	0.161	–0.077 to 0.382
HAM-A^g^	.38	0.106	–0.132 to 0.333	.96	–0.005	–0.24 to 0.23
RBD^h^	.27	0.134	–0.104 to 0.358	.22	0.15	–0.088 to 0.372
Freezing gait	.90	–0.016	–0.25 to 0.22	.94	–0.009	–0.244 to 0.226
Total UPDRS^i^	.75	0.039	–0.197 to 0.272	.58	–0.067	–0.298 to 0.17
HY^j^	.87	–0.02	–0.254 to 0.216	.74	–0.04	–0.273 to 0.196
MMSE^k^	.60	–0.064	–0.295 to 0.173	.97	–0.004	–0.239 to 0.231
NMSS^l^	.09	0.203	–0.034 to 0.418	.38	0.106	–0.132 to 0.333
PDQ39^m^	.95	–0.008	–0.242 to 0.228	.50	–0.083	–0.312 to 0.155

^a^HOG: histogram of oriented gradients.

^b^SVM: support vector machine.

^c^Tree: random forest.

^d^PD: Parkinson disease.

^e^DALEDD: levodopa equivalent daily dose.

^f^QUIP: Questionnaire for Impulsive-Compulsive Disorders in Parkinson disease.

^g^HAM-A: Hamilton Anxiety Scale.

^h^RBD: REM Sleep Behavior Disorder.

^i^UPDRS: Unified Parkinson Disease Rating Scale.

^j^HY: Hoehn & Yahr.

^k^MMSE: Mini-Mental State Examination.

^l^NMSS: Non-Motor Symptoms Scale.

^m^PDQ-39: Parkinson Disease Questionnaire-39.

### Combined Features of PD

**Figure 5:**
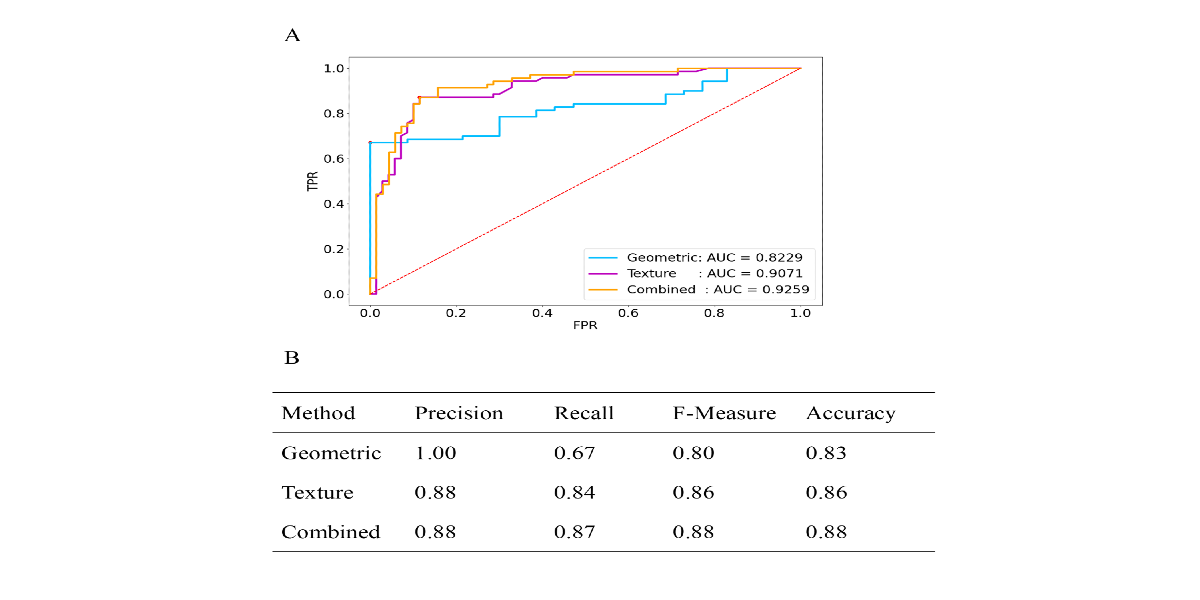
Comparison of receiver operating characteristics (ROC) analysis for the diagnosis of Parkinson disease. (A) ROC curve for each parameter and (B) result of machine learning algorithms. AUC: area under the curve; FPR: false positive rate; TPR: true positive rate.

For the combined features, it was higher (AUC 0.9259) than for geometric features alone (AUC 0.8229) and for texture features alone (AUC 0.9071), shown in Figure 5A. Similar to previous work, we obtained a Youden index as the corresponding optimal threshold from the ROC curve. Figure 5B shows that the final recognition effect of the combined features could reach 0.88, nearly 90%, with each data point as well as the comprehensive performance being improved to a certain extent.

## Discussion

### Overview

The aim of our study was to develop a computer vision–assisted AI method to extract, analyze, and recognize the facial features of patients with PD. Our results show that the accuracy of the computer vision–assisted AI method is more than 80%, and it has the advantages of timeliness, convenience, and low cost. Our results indicated that the computer vision–assisted AI method can play an important role in clinical practice for PD. First, it can analyze and summarize the facial features of patients with PD to form computer-recognizable digital information. And it can explore the representative and quantitative features of the facial features of patients with PD, which may help diagnose the disease and evaluate the effect of treatment. Second, through this method, large-scale, fast, convenient, and low-cost screening of PD in the population could be realized, helping patients who suspect PD go to the hospital as early as possible. Third, it is helpful for telemedicine and contactless medical treatment, which is of great significance during the COVID-19 pandemic. In the future, we will further improve the application of the computer vision–assisted AI method in the screening process for PD.

### Principal Findings

In this study, we used facial features combined with machine learning algorithms to distinguish PD from healthy people. First, our study confirms that the facial expressions of PD patients are different from those of healthy people, which is consistent with other studies. Second, different from the use of contact sensors to analyze facial mimicry in PD patients to achieve the purpose of identification [25], our study is contact-free and has a more profound significance for human-computer interaction. At the same time, our method is more streamlined, and the processing time is short so that we can accelerate the process of diagnosis and make it more efficient. Third, the results indicated that the combination of texture features and geometric features of PD patients could be helpful for the diagnosis of PD. Texture features show better discrimination than geometric features. What is more, the abnormal facial expression around the eyes is more pronounced than around on mouth, while the degree of abnormal facial expression was not correlated with clinically evaluated variables of PD. Our results suggest that the facial features of PD patients may be a characteristic symptom that is solely related to the disease and is not affected by the motor symptoms, nonmotor symptoms, medications, and the course of the disease. Of course, the possibility for this may come from the statistical deviation caused by an insufficient sample size and sampling error, which cannot be ignored. Further studies are necessary to confirm these findings in other neurology centers.

### Limitations

There were several limitations in our study. First, our study retrospectively enrolled treated PD patients. It is crucial to conduct prospective studies to enroll patients with de novo PD to verify the accuracy of the video of the facial feature recognition–based AI model to assist with PD diagnosis. Second, we collected videos using a single 2D video camera, and the amount of data was not sufficient. Although we were unable to capture the 3D facial expressions of the patients, 3D cameras can be used to capture more information in future research. Third, as all videos in the training data sets were classified as either PD or control, with no enrolled patients with parkinsonism, interpretation of these results cannot be extrapolated to other contexts. Facial tremor was not included as a parameter in our study for the following reasons. On the one hand, it requires expensive equipment to capture tremor information without physical contact. On the other hand, incorporating an indicator of tremor is time-consuming, because the correlation and frequency distribution characteristics between consecutive frames need to be calculated, which will greatly increase the amount of calculation required by the system. What is more, tremor greatly interferes with facial expression recognition. Although we did not involve the analysis of facial tremor in this study, we can measure it using special sensors in the future.

### Comparison With Prior Work

There was a similar study using facial features to diagnosis PD [27]. We verified the method they reported in our dataset. The results are compared with our work in Table 3. Our AI model showed better accuracy in the diagnosis of PD. Moreover, their study did not provide information on clinical characteristics of patients with PD.

**Table 3:** Comparison of results with those of prior work.

Work	Algorithm	Precision	Recall	F1 value
Jin et al [27]	SVM^a^	0.78	0.7	0.74
	RF^b^	0.6	0.9	0.72
Our method	SVM	0.8	0.87	0.84
	RF	0.88	0.87	0.88

^a^SVM: support vector machine.

^b^RF: random forest.

### Conclusions

In summary, we have verified that facial feature information is effective for distinguishing PD from MC participants. All the geometric features, texture features, and combined features had good performance. Facial features play a role in the auxiliary diagnosis of PD. A markerless 2D video, facial feature recognition–based, AI model can provide a valuable tool to assist with PD diagnosis and the potential of realizing remote monitoring especially during the COVID-19 pandemic.

## Abbreviations

AI: artificial intelligence
ANOVA: analysis of variance
AUC: area under the curve
HAM-A: Hamilton Anxiety Scale
HOG: histogram of oriented gradients
H-Y: Hoehn-Yahr
KNN: k-nearest neighbor
LBP: local binary pattern
LEDD: levodopa equivalent daily dose
MC: matched controls
MMSE: Mini-Mental State Examination
NMSS: Non-Motor Symptoms Scale
OR: odds ratio
PD: Parkinson disease
PDQ-39: Parkinson Disease Questionaire-39
QUIP: Questionnaire for Impulsive-Compulsive Disorders in Parkinson disease
RBDQ-HK: REM Sleep Behavior Disorder Questionnaire Hong Kong
RF: random forest
SVM: support vector machine
UPDRS: Unified Parkinson Disease Rating Scale
